# Neuraminidase Inhibitors in Influenza Treatment and Prevention–Is It Time to Call It a Day?

**DOI:** 10.3390/v10090454

**Published:** 2018-08-25

**Authors:** César Parra-Rojas, Van Kinh Nguyen, Gustavo Hernandez-Mejia, Esteban A. Hernandez-Vargas

**Affiliations:** Frankfurt Institute for Advanced Studies, 60438 Frankfurt am Main, Hessen, Germany; cesar.parra.r@gmail.com (C.P.-R.); admin@ytecongcong.com (V.K.N.); gustavohdezm@gmail.com (G.H.-M.)

**Keywords:** oseltamivir, neuraminidase inhibitor, influenza, mathematical modeling, epidemics

## Abstract

Stockpiling neuraminidase inhibitors (NAIs) such as oseltamivir and zanamivir is part of a global effort to be prepared for an influenza pandemic. However, the contribution of NAIs for the treatment and prevention of influenza and its complications is largely debatable due to constraints in the ability to control for confounders and to explore unobserved areas of the drug effects. For this study, we used a mathematical model of influenza infection which allowed transparent analyses. The model recreated the oseltamivir effects and indicated that: (i) the efficacy was limited by design, (ii) a 99% efficacy could be achieved by using high drug doses (however, taking high doses of drug 48 h post-infection could only yield a maximum of 1.6-day reduction in the time to symptom alleviation), and (iii) contributions of oseltamivir to epidemic control could be high, but were observed only in fragile settings. In a typical influenza infection, NAIs’ efficacy is inherently not high, and even if their efficacy is improved, the effect can be negligible in practice.

## 1. Introduction

Influenza A virus (IAV) infection affects about 20% of the worldwide population annually [1]. The 2009 influenza pandemic showed that the next pandemic could cause major adverse health and economic outcomes [2]. While vaccines remain the primary means to prevent outbreaks, the effects of influenza vaccines are mild and regularly outpaced by the antigenic drift of the virus [3]. As a result, stockpiling antiviral drugs in anticipation of a pandemic has been recommended, among other public health routines [4]. The benefits of this approach, however, have been debated in terms of treating and preventing epidemic spread [5,6].

The recommended antiviral drugs against influenza are neuraminidase inhibitors (NAIs) [7]. NAIs block the release of the influenza virus from infected host cells and thus reduce the spread of infection in the respiratory tract [8,9]. Because the influenza infection course is fast, the effects of NAIs depend on the timing of the antiviral intakes. For example, clinical trials found that oseltamivir, the most common NAI, reduced viral shedding and shortened the time to symptom alleviation by up to 1.5 days when treatment was started during the first 48 h post-infection (hpi) [5,10]. Prophylaxis with NAIs (oseltamivir, 75 mg, once or twice per day, during ten days to six weeks) was shown to be 68–90% effective in preventing infection during an influenza activity period [11,12]. Doubling the dose of oseltamivir showed no difference in outcomes [13]. By and large, the narrow time window required and the need for healthy individuals taking the drug daily for an extended period make an efficient use of NAIs questionable.

Providing concrete evidence for the drug effects from observational studies is difficult, methodologically and ethically [6]. For example, it is difficult to know the time of infection in symptomatic cases to define the drug effect. Quantifying the preventive effect is also difficult as it is not clear whether a participant was not infected or had an asymptomatic infection [14]. The long period required in the prophylaxis approach can also become problematic in terms of adherence and drug delivery. To this end, we attempted to evaluate the drug effects in a more transparent setting by using mathematical modelling and computer simulations: using a within-host infection model of influenza infection, we evaluated the efficacy of oseltamivir in general and in reducing the time to symptom alleviation; using a network model, we assessed the prophylactic effects of oseltamivir in a population and its associated cost. The analyses took oseltamivir as the example, but we expected the results to be generalizable to other NAIs.

## 2. Materials and Methods

### 2.1. Within-Host Model

**Figure 1:**
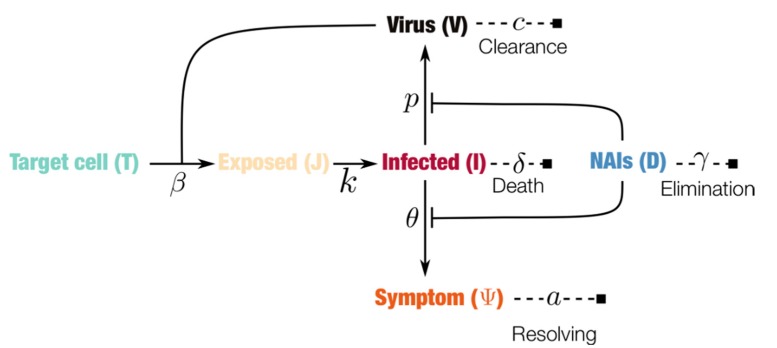
Schematic representation of the within-host influenza infection model under treatment with neuraminidase inhibitors (NAIs). Filled squares indicate endpoints, lines ending with bars indicate blocking effects and arrows indicate transitions, while dashed lines indicate the decay of the corresponding components. The system of equations and analyses are given in the Appendix A.

The influenza infection dynamics has been described with the target cell-limited model (see Figure 1) [15]. The model considers a population of epithelial cells divided into susceptible (T), latently infected (J), and productively infected (I) cells. After infection occurs at rate β, susceptible cells enter the latent phase where they remain on average 1/k days, after which they become productively infected. Infected cells shed the virus at a rate *p* and have a mean lifespan of 1/δ days. The free virus, in turn, is cleared at a constant rate *c* per day. The intensity of the symptoms, denoted by ψ, increases with the proportion of infected cells—due to the release of cytokines [16,17]—at a rate θ and has a constant resolving rate *a*. The model and parameters (see Table 1) were calibrated with human data measuring viral load and symptom score in Lukens et al. [18].

For this study, the classical sigmoid dose-response effect of NAIs was added (see the Appendix). NAIs were assumed to reduce the rate of virus shedding *p* and the symptom score. The former assumption is due to the drug blocking the release of the virus, and the latter is the result of the reduction in the host’s induction of cytokines [17]. In general, four parameters governed the effect of the NAIs: (i) the drug concentration elimination rate per day, (ii) the intake frequency (a constant interval was assumed), (iii) the dose in mg, and (iv) the concentration at which the drug reached a 50% efficacy (EC_50_). The two parameters, intake frequency and dose, defined the treatment regimen; the elimination rate and half-maximal concentration constituted the drug-specific parameters. The exploration of the sensitivity of the drug’s efficacy with respect to the above four parameters provided a complete efficacy landscape for the NAIs. The full system of equations and analytical analyses are given in the Appendix (illustrated in Figure 1).

### 2.2. Population Model

**Figure 2:**
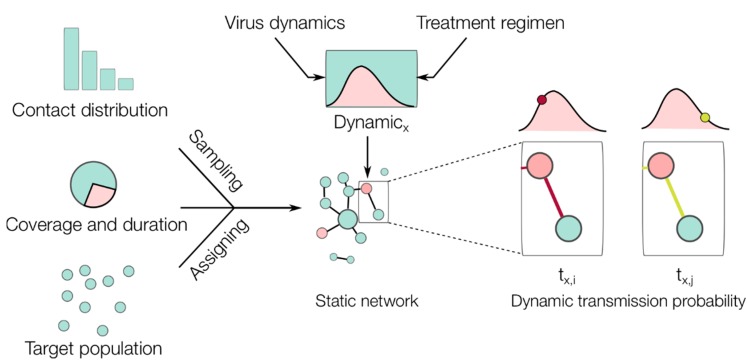
Illustration of the epidemic network model simulations. Based on empirical contact distribution data, the number of contacts (edges) was sampled and assigned to each subject (node). Based on the coverage and duration of the intervention, the nodes were assigned to either taking the drug in the defined period or not. Based on the within-host model, each infected node xth (colored red in the network) will have its own viral dynamics (red area in the dynamic) depending on whether it was already taking the drug at the time of infection or not. The transmission between infected and uninfected nodes (colored blue in the network) was evaluated in every simulation time step (e.g., i and j), during which the transmission probability varied (indicated by the edge’s color intensity) following the infection dynamics of the infected subject under consideration (see Section 2.3, Software and Algorithms, for further details).

To assess the prophylactic effects of NAIs in an epidemic context, the within-host model was used to generate the infection dynamics of an individual-based network model of influenza transmission (as illustrated in Figure 2 and detailed in Section 2.3). The following two conditions were assumed to determine the between-host transmission from the within-host dynamics: (i) the transmission potential of an infected subject at any given time is defined by its viral load at that time divided by the maximum viral load [18] (this leads to a more realistic time-dependent transmission potential based on the viral load dynamics) and (ii) the infectious period starts when the viral load crosses the threshold V_c_ = 1.35 TCID_50_/mL, as defined previously in Lukens et al. [18].

All epidemic simulations were conducted in settings that were tailored to detect the drug’s effectiveness in the models: (i) all infected individuals responded similarly to the drug (i.e., a uniform efficacy among treated individuals); (ii) uninfected individuals were equally susceptible to the infection; (iii) the drugs were assumed to be readily available and delivered to all intended recipients uniformly in time; (iv) all recipients took the drugs with complete adherence to the implemented treatment regimen; (v) all infected cases were known, including asymptomatic cases, in calculating the drug effect on reducing the epidemic size; and (vi) there were no other interventions in place and the contact network remained unchanged during the epidemic. While these conditions are unrealistic, changes observed under these conditions in the epidemic trajectory could be attributed solely to the drug’s effect.

Simulated scenarios were created based on the assumption that the interventions were constrained by a fixed amount of resources (US dollars). This was calculated based on the pandemic regimen of 150 mg oseltamivir twice daily and the minimum price for oseltamivir in large purchases: 1.6 US cents per mg as of 2006 [22]. Based on a given amount of investment, scenarios were further varied by the proportion of the population to be covered and the time during which uninfected subjects within coverage could be provided with the intended amount of drug without any disruptions. Each scenario was simulated 1000 times to obtain distributional epidemic trajectories.

### 2.3. Software and Algorithms

Open-source code (written in Python and R) is provided in a public repository for all simulations performed in this study (https://github.com/systemsmedicine/neuraminidase-inhibitors). Epidemic simulations considered a static network (10,000 nodes) that embodied the average contact distribution from the POLYMOD survey [23]. This survey asked 7290 participants in ten European countries to record their number of contacts per day, providing the most comprehensive dataset on human contact distribution to date. This empirical distribution was used to generate the static network as illustrated in Figure 2. The epidemic simulations were then carried out as follows (see Figure 2): (1) seed 100 random infected nodes; (2) find nodes connected to the infected cases and evaluate Bernoulli trials with probabilities of success depending on the viral load of the infected case, record new infected cases if any; (3) move to the next time step and repeat step 2 until no infected cases remain. Note that we initiated the epidemic with 100 infected cases to ensure that all epidemic simulations will result in many infected cases if no intervention was implemented. As such, a reduction observed in the final number of infected cases can be attributed to the effect of the intervention. For computational efficiency, the epidemic simulations were run with a one-day time step to check the transmission between the nodes. In addition, random noise was added to the time of infection by sampling from a uniform distribution U(−0.5, 0.5) in order to represent different times of contact during a given day.

## 3. Results

**Figure 3:**
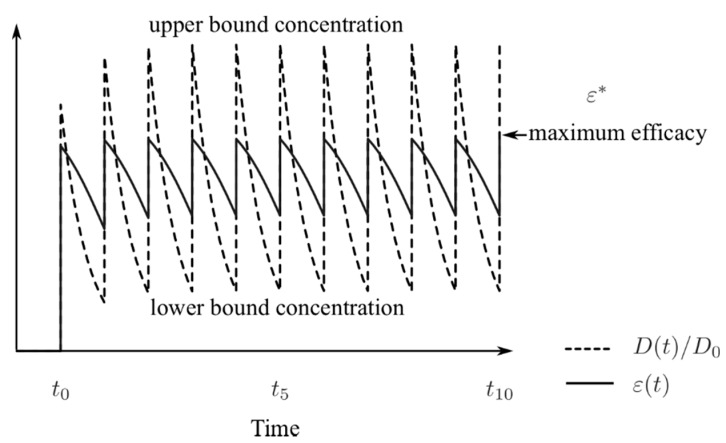
Illustration of the drug dynamics (dashed line) when administered at constant time intervals and at a fixed dose, starting at *t*_0_; the solid line is the corresponding drug efficacy. Both the drug concentration and efficacy fluctuate between their bounds between intakes.

Assuming an idealistic scenario of instantaneous absorption of NAIs, it was shown that the drug concentration stabilized to well-defined lower (*D_l_*) and upper (*D_u_*) bounds after a few doses, as illustrated in Figure 3 (see the analytical results in the Appendix). For a given drug and a given treatment regimen, *D_u_* represented a best-case scenario for the therapy where a high level of drug concentration was delivered and maintained. Correspondingly, the peak of the time-dependent drug efficacy was determined by the peak drug concentration and given by ε^*^ = *D_u_*/(*D_u_* + *EC*_50_). Thus, given a drug with an elimination rate *γ* and *EC*_50_, the correct treatment regimen was found (the dose *D*_0_ and administration interval *τ*) that yielded a desired peak efficacy with the following relationship:

(1) $$\begin{array}{rr} EC_{50} & =\left(\frac{1-\varepsilon^{*}}{\varepsilon^{*}}\right)\frac{D_{0}}{1-e^{-\gamma \tau }}. \end{array}$$

### 3.1. Reduction of Time to Symptom Alleviation

**Figure 4:**
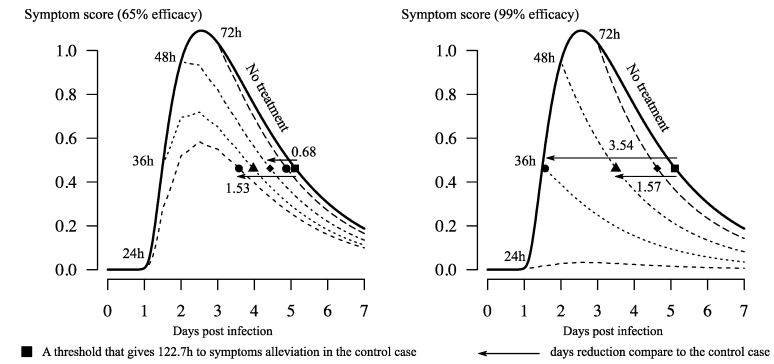
Illustration of the treatment effect on symptom score dynamics. Different shapes were used to indicate the time to first alleviation of symptoms of corresponding dynamics. The simulated treatment is 75 mg of oseltamivir taken twice daily, starting at different times post-infection; the solid line is the case without treatment. The value 122.7 h is the median of time to symptom alleviation reported in clinical trials [5]. The symptom score dynamics of the control case was taken from a previous model fitted to human volunteer data [18].

Simulations of the within-host model with the NAIs dynamics reproduced what had been observed in clinical trials (see Figure 4). Based on the symptom score dynamics of the case without treatment [18], *the time to first alleviation of symptoms* was defined as the time when the symptom score crosses a threshold that gives this measure for the control case a value of 122.7 h [5]. If the currently reported efficacy of oseltamivir is taken into consideration, the use of the defined threshold and the treatment regimen of 75 mg twice a day for five days [5] shortens the time to first alleviation of symptoms up to 1.53 days—if the drug is taken on the first-day post-infection (see Figure 4, left). Treatments initiated earlier than 48 h led to a reduction of at least more than half a day (0.68 days); the later the start of the treatment, the lesser the effect on symptom dynamics (reduction of a few hours). If a perfect drug with a similar acting mechanism is assumed, treatment started at 48 hpi could lead to an effect similar to the current drug taken at 24 hpi.

### 3.2. Inherently Limited Efficacy by Pharmacokinetic Parameters

**Figure 5:**
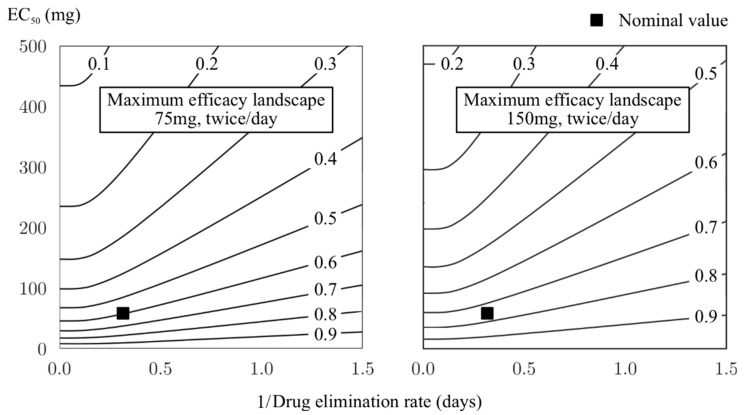
Maximum efficacy landscape of oseltamivir with respect to the drug parameters. The two recommended regimens, curative (75 mg, twice/day) and epidemic use (150 mg, twice/day), are shown. Contour lines mark the level of efficacy that can be theoretically achieved given a combination of the drug elimination rate and half-maximum concentration. The nominal value indicates the efficacy calculated based on currently reported values of the two parameters [20].

Based on the relationship between the drug and treatment parameters (Equation (1)), the pharmaceutical reported values of the drug parameters were used to explore the maximum drug efficacy that could be theoretically achieved. Figure 5 provides the landscape of oseltamivir in two treatment regimens: the curative case and epidemic cases of influenza. The former regimen provided two doses of 75 mg oseltamivir per day, whereas the latter doubled the dose to 150 mg per day. Taking into consideration the current pharmaceutical reported values for the drug [10], the maximum efficacy was approximately 60% in the curative regimen and less than 80% in the epidemic regimen. Figure 5 can also be used to find a drug with the properties yielding a desired efficacy. If the curative dose is taken into consideration, a drug with a unit elimination rate would need a half-maximal concentration no larger than approximately 10 mg to have approximately 95% efficacy or, alternatively, the current drug should have an elimination rate of approximately five times slower to have more than 70% efficacy.

**Figure 6:**
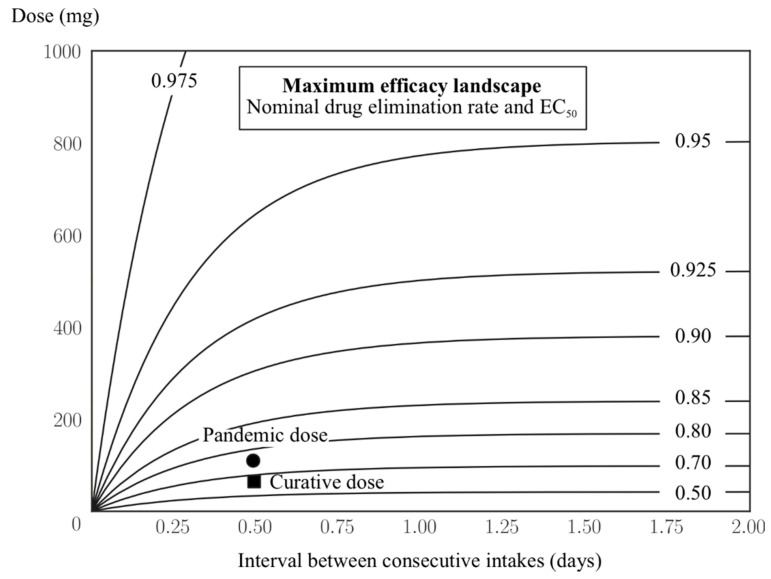
Maximum efficacy landscape of oseltamivir regarding the frequency of intakes and dose per intake. Contour lines mark the level of efficacy that can be theoretically achieved given a treatment combination. Values of the drug efficacy are shown for the two current recommended regimens.

Instead of fixing the drug pharmacokinetic parameters, a more practical way to improve the drug efficacy would be to increase either the dosage or the frequency of intakes. Figure 6 shows that for the nominal value of EC_50_ and the drug elimination rate as chosen in Table 1, a higher level of efficacy corresponds to a much larger dose or more frequent intakes than the current two recommended regimens. For example, to achieve an efficacy of 90% based on the fixed dose of 75 mg oseltamivir, one would have to take the drug more than ten times per day; instead, if the intake frequency is kept at twice per day as the recommended regimens, then a dose of approximately 300 mg would be needed. It is to be noted that these numbers are reported only for illustrating the limited efficacy of the drug and do not have a practical application, as the side-effects or toxicity when taking the drug with a higher dosage were not assessed.

### 3.3. High Preventive Effect Observed only in a Fragile Setting

**Figure 7:**
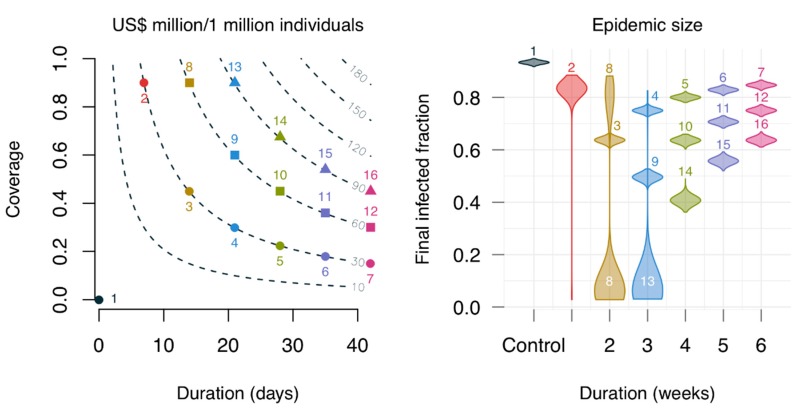
Effects of using oseltamivir on epidemic size with respect to different combinations of duration and coverage. The contour lines on the left panel show the cost in US dollars per individual, assuming the pandemic regimen (150 mg, twice daily) with 0.16 US cents per mg [22]; the numbers denote the simulated scenarios. The violin plots on the right show the epidemic size distribution for the simulated scenarios, each corresponding to 1000 simulations. Colors on both panels indicate the same simulated scenarios. The distribution in scenario 8 is bimodal, while those of scenarios 2 and 13 are skewed.

Figure 7 shows the contribution of oseltamivir to reducing the spread of an ongoing epidemic. Sixteen intervention scenarios generated based on combinations of three practical aspects of an intervention were evaluated: the allocated financial resources for stockpiling the drug, the duration during which one wishes to supply the drug to the community, and the proportion of the community upon which the interventions are going to apply. There were reductions in epidemic size for all the evaluated scenarios, which varied markedly from approximately 10% to 100%, depending on the coverage and duration of treatment. There was a trade-off between coverage and duration: prolonging the duration from one to three weeks while keeping a similarly low coverage did not bring large changes in epidemic size (scenarios 5–7, 10–12, and 14–16). However, a high coverage with a short duration (scenario 2) was not useful either as most of the simulations resulted in large epidemics. An optimal coverage duration was set between two and three weeks. Specifically, scenarios 8 and 13 (see Figure 7, bottom) both had a high coverage (90%), but the former resulted in many simulations ending with a large epidemic size (upper part), while the latter provided more certainty in controlling the epidemic. The bimodal distribution observed in scenario 8 resembled the typical pattern of the stochastic epidemic model, where the final size of the epidemic in most simulations is small and the large epidemics converge to a normal distribution [24].

## 4. Discussion

Neuraminidase inhibitors (NAIs) constitute the primary type of antiviral drugs against influenza. While the effects of the drugs have been heavily debated, it has been difficult to provide a clear assessment in observational and clinical frameworks [6]. For this paper, we used analytical and numerical analyses of a mathematical model of influenza infection to contribute to a more transparent interpretation and understanding of oseltamivir effects.

Despite using only oseltamivir as a case study, we parametrized the impact by its peak efficacy, rendering the analyses independent of the drug parameters. Specifically, while the actual value of the peak efficacy depended on the drug parameters, their functional relationship and the efficacy landscape did not. In other words, from the values of γ and EC_50_ for any given NAI, one could find its corresponding peak efficacy from the efficacy landscape defined by Equation (1). As such, the results were expected to be generalizable to other NAIs.

To show a best-case scenario for the oseltamivir, the upper bound of efficacy was used in all the analyses. This was achieved by allowing the drug concentration to converge instantaneously to its most effective level; however, in reality, this was not the case. Oseltamivir needs time to convert from oseltamivir phosphate (OP) to its active metabolite oseltamivir carboxylate (OC) [19,25]. This process depends on the rate of absorption of OP into the blood, the conversion rate from OP to OC, and the elimination rate of OC [19,26]. It was shown that taking these processes into account led to a lower upper-bound concentration of the drug, and consequently a lower efficacy than the level reported in this paper (see the Appendix). Therefore, using the instantaneous absorption model, we have already shown a better efficacy of the drug than the original reported in practice.

The simulations indicated that, for a typical case, treatments starting from 48 hpi onwards had a small effect on the symptom dynamics (see Figure 4). A treatment starting at 72 hpi resulted in a reduction of only a few hours in the time to symptom alleviation compared to the case without treatment. In an optimistic context, treatments started during the first-day post-infection would yield approximately a one-and-a-half-day reduction in the maximum time spent in symptomatic phase. This result was the main outcome reported when assessing oseltamivir effects in clinical settings [5,6,8,27]. In reality, unobservable variations of a few hours (e.g., related to infection time, treatment initiation time, and symptom-reporting time—which is subject to the reporting individual) would yield a slightly better or worse effect of the drug. The main implication is that it is highly unlikely that the drug would work as expected in practice: patients would not seek treatment without symptoms, while the typical incubation period of an influenza infection is approximately two days [7].

Limited data exist supporting the use of high-dose oseltamivir in influenza treatment; moreover, using higher doses would intensify drug shortages [28]. This study’s results revealed that the relationship between dose intake and efficacy was not linear at high levels of efficacy (see Figure 6). This result explained the small increase in efficacy when increasing oseltamivir dosage in practice [28]. Doubling the dose of oseltamivir in the pandemic regimen brings about an increase in efficacy of only approximately 10% compared to the curative regimen. To reach at least 90% efficacy (approximately 10% more than for the pandemic regimen), one might consider a dose of 500 mg twice per day; this regimen has been documented as safe [29]. The dose can be further increased, for example to 1000 mg twice per day, which results in more than 95% efficacy. However, it was noted that the current efficacy of approximately 62% of oseltamivir would still be able to attain the same net effect on the infection course if the drug were administered early enough.

Given a limited availability of the drug, as well as limited resources in the affected countries [22], the selection of prevention strategies must be well-informed. It seems that prolonging coverage over three weeks is not cost-effective (see Figure 6). This is likely to worsen in practice due to an imperfect adherence to the regimen. A best-case scenario corresponds to providing the drug for more than two weeks with as high a coverage as possible. However, this effect was observed in highly favorable conditions (see Materials and Methods), whereas several real-life conditions could compromise the drug effects. For example, the introduction of newly infected cases into the population after the coverage duration would reset the protection level of the drug. In addition, the actual drug stockpiles were very low in the 2001 H1N1 pandemic, ranging from 0.1% to 25% in rare cases [30]. Nevertheless, in a small community, the results could hold if epidemic prevention routines are in place [31] (e.g., examining newcomers, providing quarantine and isolation measures, closing schools, and reducing social activities). The effect of the drug, in this case, is very difficult to discern. A more realistic approach is an unequal distribution of the drug in order to target highly transmissible subgroups of the population offered in tandem with public health routines [31].

This study presented the following limitations: (i) the within-host model lacked a marker for mortality and, as a result, the effects of oseltamivir in reducing the risk of death could not be assessed, as suggested elsewhere [5]; (ii) the effects of the drug on viral load and symptom severity were assumed to be the same. However, if the same functional form was taken into consideration in both cases, the reduction in the production of cytokines was expected to be milder than the main effect of the drug-inhibiting virus shedding, so that this simplification could be considered as part of the best-case scenario assumption; and (iii) assessments of coverage and duration did not consider the potential emergence of drug-resistance influenza strains, which in effect might suggest a different strategy. For example, intermediate levels of coverage could be preferred to very high ones to keep both drug-sensitive and drug-resistant strains at bay [31]; this again can be interpreted as a best-case scenario.

## 5. Conclusions

Oseltamivir works, but with a limited efficacy that is constrained by its intrinsic pharmacokinetic parameters and influenza host dynamics. If oseltamivir is used as a therapeutic measure, the delay from the time of infection to the time of treatment initiation leads to the underperformance of the drug. While the efficacy could be increased by increasing dosage, a drug with 99% efficacy administered after 48 hpi yields a negligible effect on influenza virus dynamics and symptoms. If oseltamivir is used as a prophylactic measure, the drug effectiveness is only warranted under limited conditions and should only be considered as an expensive alternative to public health routines in epidemic control.

## Figures and Tables

**Table 1:** Parameter values used in the simulations. The initial conditions corresponded to a susceptible cell population T(0) = 1 and an inoculum V(0) = 7.5 × 10^−6^ TCID_50_ mL^−1^.

Parameter	Value	Unit	Source or Notes
β	0.0674	TCID_50_ mL day^−1^	[18]
*k*	3.684	day^−1^	[18]
δ	1.364	day^−1^	[18]
*p*	40,356	TCID_50_ mL^−1^ day^−1^	[18]
*c*	8.0	day^−1^	[18]
θ	2.75	day^−1^ S ^*^	[18]
*a*	0.498	day^−1^	[18]
γ	3.26	day^−1^	[19]
EC_50_	0.01–500 ^†^	mg	[20]
D_0_	75 (c) and 150 (p) ^**^	mg	[21]
τ	0.5	day	twice a day

TCID_50_: 50% Tissue culture Infective Dose, * the symptoms score units; ** the curative (c) and pandemic (p) dosage regimen. ^†^ Consider the half-maximal concentration ranging from approximately EC_50_ = 0.0008 µM to 35 µM, with 1 µM ≈ 0.284 mg/mL and a volume of distribution for oral administration of approximately 50 L [20].

**Figure A1:**
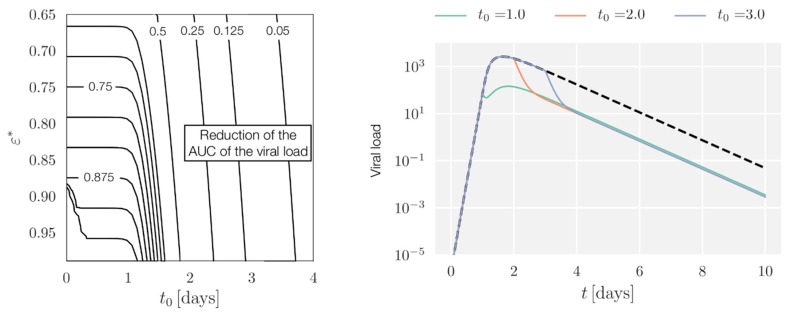
Left: Contour lines show the fraction of reduction in the area under the curve (AUC) of the viral load dynamics as a function of treatment initiation times and peak drug efficacy. Right: Dynamics of the within-host system in the case without treatment (black, dashed lines) and in the case with treatment for ε* = 0.95 and different therapy starting times (colored lines). To avoid artificial growth of the virus late after infection, simulations with a viral load below a prescribed tolerance–in this case, 10^−3^ TCID50 mL^−1^–one-day post-infection are assumed to have stopped growing. In all cases, the treatment corresponds to the curative regimen for oseltamivir: D_0_ = 75 mg, τ = 0.5 days.

## Appendix A

### Appendix A.1. Model Analyses

The within-host infection model equations are as follows:

(A1) $$\begin{array}{l} \dot{T}=-\beta \mathrm{TV},\;\\ \dot{J}=\beta \mathrm{TV}-\mathrm{kJ},\\ \dot{I}=\mathrm{kJ}-\delta I,\\ \dot{V}=p\left(1-\varepsilon \right)I-\mathrm{cV},\\ \dot{\Psi }=\theta I\left(1-\varepsilon \right)-a\Psi ,\\ \dot{D}=-\gamma D,\;t_{k}<t<t_{k+1.} \end{array}$$

The quasi-steady states of the drug effect are found by noting that, when the therapy is started:

(A2) $$\;D\left(t_{0}^{-}\right)=0,\;D\left(t_{0}^{+}\right)=D_{0}.$$

After a time τ, the initial value of D will have decayed by an amount equal to $e^{-\gamma \tau }$, so that the drug concentration just before and just after the second administration will be given by:

(A3) $$\;D\left(t_{1}^{-}\right)=D_{0}e^{-\gamma \tau },\;D\left(t_{1}^{+}\right)=D_{0}e^{-\gamma \tau }+D_{0},$$

where the interval between intakes is constant and equal to τ. Repeating this reasoning several times, and when time $t_{n}$ is reached, the drug concentration has the following form:

(A4) $$\;\begin{array}{ll} D\left(t_{n}^{-}\right) & =D_{0}e^{-\gamma \tau }+D_{0}e^{-2\gamma \tau }+\ldots +D_{0}e^{-n\gamma \tau }=D_{0}\overset{n}{\underset{k=1}{\sum }}e^{-k\gamma \tau },\\ D\left(t_{n}^{+}\right) & =D_{0}+D_{0}e^{-\gamma \tau }+D_{0}e^{-2\gamma \tau }+\ldots +D_{0}e^{-n\gamma \tau }=D_{0}\overset{n}{\underset{k=0}{\sum }}e^{-k\gamma \tau }. \end{array}$$

The summations in the expressions above have a closed form when n→∞, provided that γτ>0 [32], which is naturally the case since both parameters are positive. Therefore, the lower and upper bounds of the drug concentration are obtained as follows:

(A5) $$\;\begin{array}{rr} D_{l}\; & =\frac{D_{0}e^{-\gamma \tau }}{1-e^{-\gamma \tau }},\\ D_{u} & =\frac{D_{0}}{1-e^{-\gamma \tau }}; \end{array}$$

these are reproduced in Section 3 of the main text.

A similar approach can be used to obtain the values to which the peaks and valleys of the drug concentration converge after much time has passed for the more complicated model found in [25]. These lower and upper bounds are given by the following:

(A6) $$\;\begin{array}{rr} D_{l}^{\prime }\; & =D_{0}K_{1}\left[\frac{1}{1-\exp\left(-k_{e}\tau \right)}-\frac{1}{1-\exp\left(-k_{a}\tau \right)}\right],\\ D_{u}^{'} & =D_{0}K_{1}\left[\frac{K^{K_{2}}}{1-\exp\left(-k_{e}\tau \right)}-\frac{K^{K_{1}}}{1-\exp\left(-k_{a}\tau \right)}\right], \end{array}$$

where $k_{a}$ and $k_{e}$ are, respectively, the absorption and elimination rates of the drug, and

(A7) $$\;\begin{array}{ll} K_{1} & =\frac{k_{a}}{k_{a}-k_{e}},\\ K_{2} & =\frac{k_{e}}{k_{a}-k_{e}},\\ K & =\frac{k_{a}\left[1-\exp\left(-k_{e}\tau \right)\right]}{k_{e}\left[1-\exp\left(-k_{a}\tau \right)\right]}. \end{array}$$

The result is that $D_{u}>D_{u}^{\prime }$, and a simpler description is therefore presented in the main text.

### Appendix A.3. AUC Reduction with Full Pharmacokinetic-Pharmacodynamic (PK/PD)

So far, it was assumed that the NAI was fully and instantaneously absorbed, and that a more realistic approach would have to consider the rate of absorption. In the case of oseltamivir, one must also consider the conversion from oseltamivir phosphate (OP) to its active metabolite, oseltamivir carboxylate (OC); this can be described by a two-compartment model for OP and OC [19,27].

Following [28], we explored the within-host model, and the equations for the drug dynamics were replaced by:

(A8) $$\;\begin{array}{ll} \dot{D} & =-k_{a}D\;\\ \dot{\mathrm{OP}} & =k_{a}D-k_{f}\mathrm{OP}\\ \dot{\mathrm{OC}} & =k_{f}\mathrm{OP}-k_{e}\mathrm{OC}\\ \varepsilon \left(t\right) & =\frac{\mathrm{OC}\left(t\right)}{\mathrm{OC}\left(t\right)+{\mathrm{EC}}_{50}}, \end{array}$$

where $k_{a}$, $k_{f}$, and $k_{e}$ represented, respectively, the rate of absorption of OP into the blood, the conversion rate from OP to OC, and the elimination rate of OC. In order to be consistent with the parametrization, the values of $k_{a}$, $k_{f}$, and $k_{e}$ were fixed, respectively, at $k_{a}=24.24\;{\mathrm{days}}^{-1}$, $k_{f}=16.41\;{\mathrm{days}}^{-1}$, and $k_{e}=3.26\;{\mathrm{days}}^{-1}$ [19], and ${\mathrm{EC}}_{50}$ was obtained from Equation 1 (see the main text) for a given $\varepsilon^{*}$, noting that in this case $k_{e}=\gamma$. The resulting outcomes were slightly worse in terms of effectiveness.
